# Potential for misclassification of community-acquired respiratory virus infections as healthcare-associated respiratory virus infections at a pediatric healthcare system

**DOI:** 10.1017/ice.2026.10450

**Published:** 2026-06

**Authors:** Zachary M. Most, Trish M. Perl, Michael Sebert

**Affiliations:** 1 Division of Pediatric Infectious Disease, Department of Pediatrics, University of Texas Southwestern Medical Center, Dallas, TX, USA; 2 Peter O’Donnell School of Public Health, Dallas, TX, USA; 3 Division of Infectious Diseases and Geographic Medicine, Department of Internal Medicine, University of Texas Southwestern Medical Center, Dallas, TX, USA

## Abstract

During a period of universal admission respiratory virus testing, many events (5%–14%) that might have been classified as healthcare-associated respiratory viral infections (HARVI) during routine operations were found to be community-acquired. These findings emphasize unique challenges for HARVI surveillance and the impact that testing strategies have on reported rates.

## Introduction

Healthcare-associated (HA) respiratory virus infections (HARVI) are a common source of patient morbidity in pediatric hospitals.^1–3^ Surveillance strategies and definitions for HARVIs have not been standardized, and methods typically used by the National Healthcare Safety Network to discriminate HA from present-on-admission infections do not optimally identify HARVIs because of longer incubation periods, variable incubation periods among viruses, frequency of asymptomatic infections, and prolonged asymptomatic shedding of viral nucleic acids after an acute infection.^4–9^ It is plausible that patients may acquire infections in the community, be incubating these infections at the time of admission, and then show symptoms and test positive only later in the admission. These events are not preventable in the healthcare setting and confound HARVI surveillance and prevention strategies. During the COVID-19 pandemic, patients at our pediatric healthcare system were screened on admission for SARS-CoV-2 and other respiratory viruses using polymerase chain reaction (PCR) or antigen tests. This provided a unique opportunity to assess the presence of respiratory viruses upon hospital admission and examine subsequent symptom development and clinical testing results later in the admission. We describe the unintended impact that this screening program had on reducing misclassification of community-acquired respiratory infections as HARVIs.

## Methods

All patients who tested positive for a respiratory virus on hospital day 3 or later while admitted to one of three hospitals in a quaternary-care pediatric healthcare system (490 beds, 72 beds, 38 beds) in North Texas from August 2, 2020, through April 17, 2022, were included. This retrospective cohort corresponded to the period of universal admission respiratory virus testing with required documentation of symptom status. When placing the order, the provider recorded the patient’s symptom status in the electronic health record (Supplemental Methods 1). For this study, we defined an ‘admission screen’ as the first PCR or antigen test for at least one respiratory virus that was collected within one calendar day of admission. Subsequent respiratory virus testing on a hospitalized patient may have been performed due to the onset of new symptoms or to remove isolation precautions, but was at the discretion of the clinician (Supplemental Methods 2).

For this analysis, each positive test from hospital day 3 or later was assigned to an ‘onset category’: 1. *community-onset window* (CO): test collected on a hospital day prior to the minimum incubation period for the specific virus, 2. *possible HA window*: test collected on a hospital day between the minimum and maximum incubation periods for the specific virus, and 3. *definite HA window*: test collected on a hospital day after the maximum incubation period for the specific virus (Supplemental Table 1 and Supplemental Figure 1).^6,7^ Positive tests were further categorized as repeat positives or first positives. To evaluate the impact of admission screening on positive respiratory virus test results, we compared the frequency of positive tests during the study period to an interval without universal admission testing (Jan-Dec 2023). The data were then cross-referenced with infection prevention and control surveillance data on definite HARVIs during the universal testing period. Our surveillance definition for HARVIs included a provision that any repeat positive tests for the same virus during the same admission were excluded (Supplemental Methods 3). Each onset category was described by demographics, presence or absence of respiratory symptoms on admission, and results of admission screen. Within the repeat positives in the definite HA window, we explored the hypothetical scenario to determine how many surveillance HARVIs would have been identified under routine operations without universal admission screening. This was based on provider documentation of symptoms on admission and repeat testing, and review of medical records to determine if repeat testing was likely to have been classified a HARVI without admission testing (Supplemental Methods 4).

## Results

**Figure 1.**
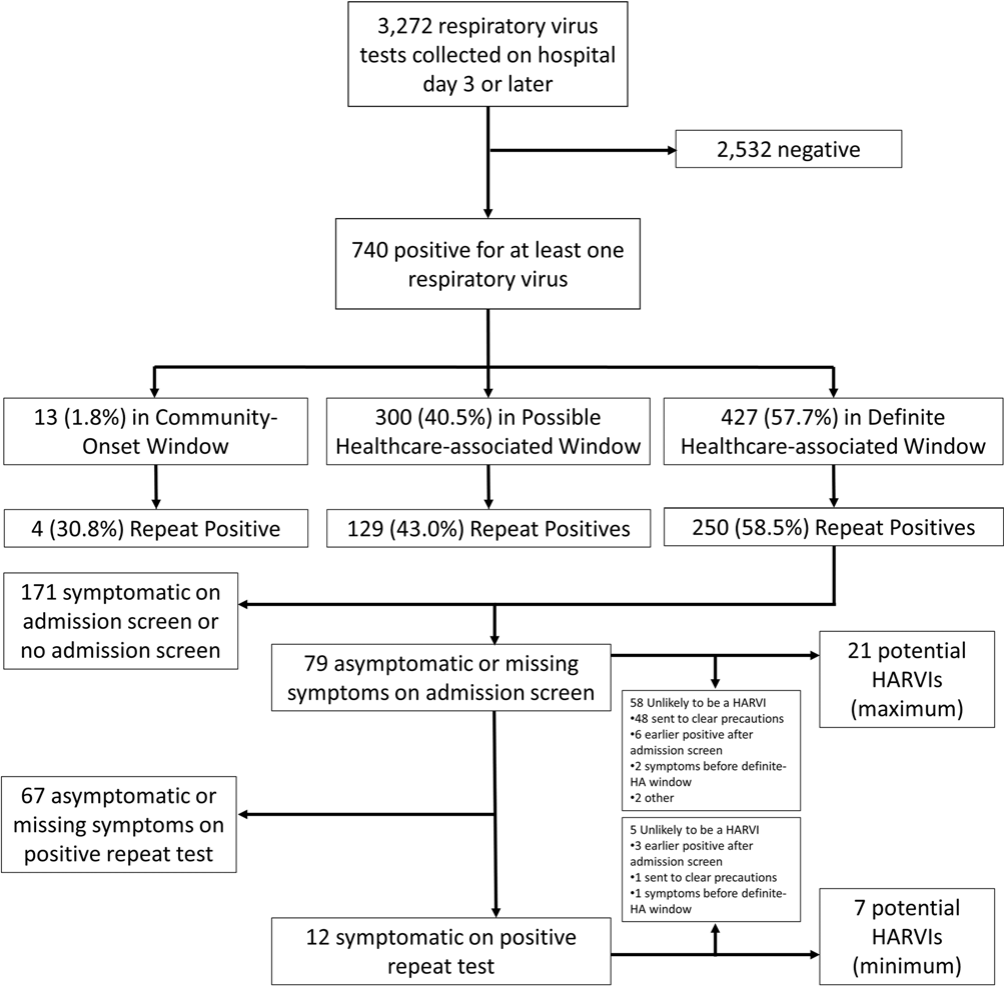
Figure 1 long description. Flowchart showing respiratory virus test results on hospitalized patients on hospital day 3 or later from August 2, 2020 to April 17, 2022. See text for definition of the onset windows and Supplemental Methods 4 for details on how the maximum and minimum potentially misclassified HARVIs were calculated. The other category included an asymptomatic child who had a COVID-19 exposure and an asymptomatic child with parental request for COVID-19 test. HARVI, healthcare-associated respiratory virus infection; HA, healthcare-associated.

During the study period, 3,272 respiratory virus tests were collected on or after the 3^rd^ hospital day, and 740 (22.6%) detected at least one virus (Figure 1). Most tests were performed in young children on the general pediatrics floors (Table 1). When compared to a period without universal screening, a greater proportion of positive tests obtained in the CO and possible HA windows were repeat positives, but the proportions were similar for tests obtained in the definite HA window (Supplemental Table 2). Patients with repeat positive tests were much less likely to have an admission screen missing in the electronic health record and were more likely to be symptomatic at the time of admission screening (Supplemental Table 3). Of the 177 positive tests obtained in the definite HA window that were not repeat positives, our surveillance program classified 125 (71%) as HARVIs. The remaining 52 (29%) were not classified as HARVIs due either to a lack of symptoms or symptom onset that occurred during the possible HA or CO window.

**Table 1.** Demographic characteristics of patients tested for a respiratory virus on hospital day 3 or later Table 1 long description.

Characteristic	Category	Tested on hospital day 3 or later (n = 3,272)	Positive on hospital day 3 or later (n = 740)
Age, median (IQR), years		2 (0–11)	3.5 (1–11)
Sex, n (%)	Female	1,534 (46.9)	364 (49.2)
	Male	1,737 (53.1)	376 (50.8)
	Unknown	1 (0.0)	0
Unit, n (%)	Floor	2,789 (85.2)	655 (88.5)
	ICU	483 (14.8)	85 (11.5)
Type of test, n (%)	PCR panel w/SARS-CoV-2	1,870 (57.2)	456 (61.6)
	PCR panel w/o SARS-CoV-2	898 (27.4)	187 (25.3)
	SARS-CoV-2/Influenza/RSV PCR	142 (4.3)	58 (7.8)
	SARS-CoV-2 only	362 (11.1)	39 (5.3)
Hospital day of test (admission = day 1)	3 to 4	439 (13.4)	126 (17.0)
	5 to 7	467 (14.3)	110 (14.9)
	8 to 14	729 (22.3)	204 (27.6)
	15+	1,637 (50.0)	300 (40.5)
Onset class	CO window	–	13 (1.8)
	Possible HA window	–	300 (40.5)
	Definite HA window	–	427 (57.7)

PCR panel — BIOFIRE respiratory panel (Biomerieux, Marcy-l’Etoile France).

ICU, intensive care unit; PCR, polymerase chain reaction; SARS-CoV-2, severe acute respiratory syndrome coronavirus 2; RSV, respiratory syncytial virus; CO, community-onset; HA, healthcare-associated.

A total of 79 repeat positive tests were collected during the definite-HA window from patients who were asymptomatic or had missing symptom status on admission screening, 12 (15%) of these were subsequently reported as symptomatic on their repeat positive test (Figure and Supplemental Table 4). Based on chart review of indication for repeat testing, we estimated that under routine operations without universal screening, between 7 and 21 of these positive tests would have been classified as HARVIs. Under this hypothetical, between 5.3% and 14.4% of HARVIs would represent community-acquired infections that were misclassified as healthcare-associated.

## Discussion

In this study, we found that in a hypothetical scenario without universal admission screening for respiratory viruses during the COVID-19 pandemic, up to 14% of patients who met surveillance criteria for a HA respiratory virus infection actually had community-acquired infections. There are two important conclusions to draw from these results. First, even when using a definition that requires new onset symptoms and accounts for long viral incubation periods, a meaningful number of cases that meet surveillance definitions for HARVIs will actually be community-acquired infections that are misclassified. This is likely due to prolonged shedding of viral nucleic acids combined with the lack of specificity of upper respiratory symptoms or fever as clinical indicators. Second, measured HARVI incidence depends on testing strategies for respiratory viruses (such as universal admission testing and how often diagnostic testing is used for respiratory infections). Therefore, it is difficult to compare HARVI incidence between different institutions and time periods. The large volume of repeat testing at our institution may have little clinical or infection control benefit and is an ongoing target for diagnostic stewardship intervention. Limitations of this study include possible variability in testing strategies for respiratory virus infections in hospitalized patients during the study period, the possibility that ‘repeat positive’ test results for an identical virus indeed reflect a newly hospital-acquired infection with the same virus rather than a misclassified community-onset infection, and that symptom status as determined by the ordering provider was intended to capture symptoms of SARS-CoV-2 infection and not respiratory virus infections in general.^10^ The role of respiratory infections in patient safety is increasingly recognized; however their use as a quality indicator is problematic, as more than one in seven HAIs by surveillance definition may actually be misclassified community-acquired infections, even with conservative surveillance definitions. While it remains critical to prevent HARVIs in healthcare settings, accurate surveillance strategies remain elusive.

## Supporting information

10.1017/ice.2026.10450.sm001 Most et al. supplementary material Most et al. supplementary material

## Supplementary material

The supplementary material for this article can be found at https://doi.org/10.1017/ice.2026.10450.

## Figure 1 Long description

The flowchart begins with 3,272 respiratory virus tests collected on hospital day 3 or later. Out of these, 2,532 tests were negative, and 740 were positive for at least one respiratory virus. The positive cases are divided into three categories: 13 (1.8%) in the Community-Onset Window, 300 (40.5%) in the Possible Healthcare-associated Window, and 427 (57.7%) in the Definite Healthcare-associated Window. Each category further breaks down into repeat positives: 4 (30.8%) in the Community-Onset Window, 129 (43.0%) in the Possible Healthcare-associated Window, and 250 (58.5%) in the Definite Healthcare-associated Window. The flowchart then categorizes patients based on symptoms: 171 symptomatic on admission screen or no admission screen, 79 asymptomatic or missing symptoms on admission screen, and 67 asymptomatic or missing symptoms on positive repeat test. Among the asymptomatic or missing symptoms on admission screen, 21 are potential HARVIs (maximum), with 58 unlikely to be HARVIs due to various reasons. Among the 12 symptomatic on positive repeat test, 7 are potential HARVIs (minimum), with 5 unlikely to be HARVIs due to various reasons.

Navigate back to Figure 1.

## Table 1 Long description

The table presents demographic characteristics of patients tested for a respiratory virus on hospital day 3 or later. It includes data on age, sex, unit, type of test, hospital day of test, and onset class. The table has 11 rows and 5 columns. Key columns include characteristics, category, tested on hospital day 3 or later, and positive on hospital day 3 or later. Notable trends include a higher percentage of males tested and positive, most tests conducted on the floor rather than in the ICU, and a significant number of tests performed using the PCR panel with SARS-CoV-2. The data also shows that a substantial proportion of positive tests were conducted on hospital day 15 or later.

Navigate back to Table 1.
