# Supplementary material for: Potential for misclassification of community-acquired respiratory virus infections as healthcare-associated respiratory virus infections at a pediatric healthcare system
Source: Infect Control Hosp Epidemiol. 2026 Apr 10;47(6):626–9. doi: 10.1017/ice.2026.10450 (PMC13216798; doi:10.1017/ice.2026.10450)
Supplement: Most et al. supplementary material [file S0899823X26104504sup001.docx]

Supplement to: Potential for misclassification of community-acquired respiratory virus infections as healthcare-associated respiratory virus infections at a pediatric healthcare system

**Supplemental Methods 1**

Determination of symptom status

Symptom status was determined by the provider who ordered the respiratory panel in an electronic order using the Epic electronic health record (Epic Systems Corporation, Verona, WI). The order-set asked the user if the patient had symptoms of COVID-19 using the phrase “Is the patient symptomatic as defined by CDC”. The user could select “Yes”, “No”, or “Unknown”. The Centers for Disease Control and Prevention (CDC) definition of COVID-19 symptoms included: fever or chills, cough, shortness of breath or difficulty breathing, fatigue, muscle or body aches, headache, new loss of taste or smell, sore throat, congestion or runny nose, nausea or vomiting, and diarrhea.

In a prior study we evaluated the accuracy of this selection with regards to any URI symptom in the patient (see Most ZM et al. Respiratory virus infections in symptomatic and asymptomatic children upon hospital admission: new insights. ASHE. 2024). Using chart reviewer designation as the gold standard, the ordering provider selection had 60.9% sensitivity and 96.3% specificity for respiratory symptoms or fever in the patient.

**Supplemental Methods 2**

Isolation precautions and removal of isolation precautions at the study location

Throughout the study period at our pediatric healthcare system, the isolation precautions required by our infection prevention and control policy for respiratory viruses other than SARS-CoV-2 remained unchanged. Patients with suspected or laboratory-confirmed respiratory virus infections were required to be placed on contact and droplet precautions (in addition to standard precautions), even if they were asymptomatic. Patients were required to remain on contact and droplet precautions for the duration of illness, AND until fever resolved for at least 24 hours, AND until having one negative respiratory virus PCR test. Infection preventionists did not routinely remind clinicians to perform testing to discontinue isolation. The last criterion led to many patients getting a repeat respiratory virus PCR panel in order to try to remove the patient from isolation precautions.

Isolation precautions for SARS-CoV-2 infection changed throughout the study period. ‘Enhanced Contact/Droplet’ precautions were required for patients with suspected or laboratory-confirmed SARS-CoV-2 infection, which included use of gown, gloves, an N95 or equivalent respirator protection, and eye protection. Clearance criteria from isolation precautions for SARS-CoV-2 infection were in accordance with US CDC recommendations. Immunocompetent patients with mild to moderate illness were required to remain on isolation precautions until fever resolved for at least 24 hours without use of antipyretics, other symptoms had improved, AND 10 days had passed from the onset of symptoms (without a requirement for repeat PCR testing).

During the period of mandatory admission testing for SARS-CoV_2, asymptomatic patients were not required to be placed on contact or droplet precautions pending test results. If positive, such precautions were started and continued until clearance criteria were met.

**Supplemental Methods 3**

Surveillance definition for healthcare-associated respiratory virus infection (HARVI)

1. Microbiologic criterion: an upper or lower respiratory specimen testing positive on an antigen test (Quidel Sofia^®^ Influenza A+B FIA, Quidel Sofia^®^ RSV FIA, or Quidel Sofia^®^ Flu+SARS Antigen FIA [Quidel Corporation, San Diego, California]) or a respiratory pathogen multiplex polymerase chain reaction (PCR) panel (BioFire^®^ FilmArray^®^ Respiratory Panel, BioFire^®^ FilmArray^®^ Respiratory Panel 2.1 [bioMérieux, Marcy-l'Étoile, France], Xpert Xpress influenza/RSV PCR, or Xpert Xpress Cov-2/influenza/RSV PCR [GeneXpert^®^ Dx system, Cepheid^®^, Sunnyvale, California]) for one of the following viruses: adenovirus, common cold human coronavirus (HKU1, NL63, 229E, or OC43), human metapneumovirus, influenza A (H1N1pdm2009 or H3N2), influenza B, human parainfluenza virus (type 1, 2, 3, or 4), respiratory syncytial virus (RSV), rhinovirus/enterovirus (which are not distinguished on the panel), or SARS-CoV-2;

2. Symptomatic criterion: at least one new sign or symptom associated with a lower or upper respiratory infection following hospital admission, including conjunctivitis, cough, hypoxia, increased endotracheal secretions, increased ventilator settings, increased work of breathing, nasal congestion, rhinorrhea, sneezing, or tachypnea;

3. Chronologic criterion: the onset of symptoms was on or after a minimum number of days from hospital admission for each specific virus listed below. Minimum intervals were defined as being one day longer than the upper limit of the incubation period for each virus described in the American Academy of Pediatrics Red Book (for viruses except SARS-CoV-2). See Supplemental Table 1 for specific details.

Only the first positive test for a specific virus is eligible for a HARVI. All repeat positive tests during the index admission are excluded from HARVI surveillance.

The date of event for HARVI surveillance is the earlier date that criterion 1 or 2 was met. In the present manuscript, we describe ‘possible HA window’ and ‘definite HA window’ as new terms to distinguish these from the surveillance definition of ‘HARVI’. The surveillance definition of HARVI required the presence of new signs or symptoms, but the ‘windows’ evaluated in this manuscript looked only at date of testing.

**Supplemental Methods 4**

Method of calculation of range of potential surveillance HARVIs averted due to universal admission screening

Our hospital’s isolation precautions rules for patients with confirmed respiratory virus infections required a negative PCR test (among other criteria) to be removed from those isolation precautions. Many patients who tested positive for a respiratory virus on an admission screen may have had subsequent PCR testing done only to remove them from isolation precautions. This subsequent test likely would not have been done in the absence of universal admission screening. Therefore, the introduction of admission screening itself likely increased the number of follow-up PCR tests that were done. Additionally, our surveillance definition for HARVIs uses symptom onset as the date of event, so patients who were tested during the ‘Definite HA window’ but had symptom onset during the ‘Possible HA window’ would not be counted as HARVIs using our surveillance definition. We attempted to adjust for these issues using the following method.

We started with the number of repeat positives for any respiratory virus in the definite HA window with positive admission screen who were asymptomatic or had missing symptoms on admission screen (N =79) and the subset of those who were symptomatic on their repeat positive testing (N = 12). These were the initial range of tests that may have been misclassified as HARVIs under the hypothetical scenario of no admission testing. We then performed a chart review of these 79 patients to determine if their repeat test fell into either of the following two categories: 1) Collected solely for the purpose of attempting to remove a patient from isolation precautions, or 2) Had symptom onset in the possible HA window.

We determined that of the 79 patients mentioned above, 58 of them were unlikely to have been HARVIs according to our surveillance infection due to:

- 48 of them had their repeat test done without any new symptoms and done to remove the patient from isolation precautions,
- 6 had another test done that was collected after the admission screen and before the test in question, with symptoms at the time of the earlier test, and tested positive for the same virus,
- 2 had symptom onset that occurred prior to definite-HA window,
- 1 was asymptomatic and tested due to a COVID-19 exposure, and
- 1 was asymptomatic and tested due to parental request. This left us with a maximum estimate of potentially misclassified HARVIs of 21.

For the minimum estimate we only included children who were reported as symptomatic on their repeat positive test. Out of the 12 patients, 7 of them were likely to have been classified as HARVIs without admission testing, which was our minimum estimate (range 7 to 21).

| **Supplemental Table 1**: Hospital-day windows for classification of first positive test for each virus | | | |
| --- | --- | --- | --- |
|  | **Minimum hospital day of testing** | | |
| **Virus** | **CO Window** | **Possible HARVI Window** | **Definite HARVI Window** |
| Adenovirus | 1-4 | 5-14 | ≥ 15 |
| Coronavirus | 1-2 | 3-5 | ≥ 6 |
| Metapneumovirus | 1-3 | 4-5 | ≥ 6 |
| Influenza A | 1-2 | 3-4 | ≥ 5 |
| Influenza B | 1-2 | 3-4 | ≥ 5 |
| Parainfluenza | 1-2 | 3-6 | ≥ 7 |
| RSV | 1-3 | 4-6 | ≥ 7 |
| Rhino/enterovirus | 1-2 | 3-7 | ≥ 8 |
| SARS-CoV-2 | 1-2 | 3-10 | ≥11* |
| CO – community onset; HARVI – healthcare-associated respiratory virus infection; SARS-COV-2 – severe acute respiratory syndrome coronavirus 2  * For SARS-CoV-2, The minimum hospital day of symptom onset for a definite HARVI was hospital day 15 from March 2020 to December 2021, then was changed to hospital day 11 after the emergence of the omicron variant characterized by a shorter incubation period.  Reference: Lessler J, Brookmeyer R, Reich NG, Nelson KE, Cummings DA, Perl TM. Identifying the probable timing and setting of respiratory virus infections. *Infect Control Hosp Epidemiol* 2010;31:809-815. | | | |

| **Supplemental Table 2.** Comparison of onset categories during universal screening period and non-screening period | | | | | |  |  |  |
| --- | --- | --- | --- | --- | --- | --- | --- | --- |
|  | Universal screening period (Aug 2020 - Apr 2022) N = 740 | | Non-screening period (Jan 2023 - Dec 2023) N = 642 | |  |  |  |  |
| Onset Category | n | % | n | % |  |  |  |  |
| CO window | 13 | 1.8% | 19 | 2.8% |  |  |  |  |
| Repeat Positive | 4 | 30.8% | 0 | 0.0% |  |  |  |  |
| Possible HA window | 300 | 40.5% | 214 | 31.5% |  |  |  |  |
| Repeat Positive | 129 | 43.0% | 3 | 1.4% |  |  |  |  |
| Definite HA window | 427 | 57.7% | 447 | 65.7% |  |  |  |  |
| Repeat Positive | 250 | 58.5% | 242 | 54.1% |  |  |  |  |
|  |  |  |  |  |  |  |  |  |
| Only includes positive tests for any respiratory virus on hospital day 3 or later. The ‘Repeat Positive’ category is only repeat positive tests within the onset category above, and the denominator for the percentage is the number of positive tests in that onset category. CO – community onset, HA - healthcare-associated | | | | | |  |  |  |

| **Supplemental Table 3**. Demographic and testing characteristics compared between onset categories for those who tested positive for a respiratory virus on hospital day 3 or later (N=740) during a period of universal admission screening (Aug 2020 – Apr 2022). | | | | | | | | | |
| --- | --- | --- | --- | --- | --- | --- | --- | --- | --- |
| Characteristic | Category | CO Window (N=14) | Possible HA Window (N=300) | Definite HA Window (N=427) | P | Definite HA Window First Positive (N=177) | Definite HA Window Repeat Positive (N=250) | P |  |
| Age, median (IQR), years |  | 2 (1-3) | 4 (1-12) | 3 (1-11) | 0.03 | 2 (1-7) | 4 (1-12) | 0.02 |  |
|  |  |  |  |  |  |  |  |  |  |
| Sex, n (%) | Female | 10 (77%) | 139 (46%) | 215 (50%) | 0.07 | 89 (50%) | 126 (50%) | 0.98 |  |
|  | Male | 3 (23%) | 161 (54%) | 212 (50%) |  | 88 (50%) | 124 (50%) |  |  |
|  |  |  |  |  |  |  |  |  |  |
| Unit, n (%) | Floor | 12 (92%) | 277 (92%) | 366 (86%) | 0.02 | 157 (89%) | 209 (84%) | 0.14 |  |
|  | ICU | 1 (8%) | 23 (8%) | 61 (14%) |  | 20 (11%) | 41 (16%) |  |  |
| Symptomatic on admission screen | Yes | 6 (46%) | 106 (35%) | 136 (32%) | 0.08 | 31 (18%) | 104 (42%) | 0.01 |  |
|  | No | 3 (23%) | 98 (33%) | 177 (41%) |  | 75 (42%) | 101 (40%) |  |  |
|  | Missing symptom status | 0 (0%) | 15 (5%) | 30 (7%) |  | 14 (8%) | 16 (6%) |  |  |
|  | No admission test | 4 (31%) | 81 (27%) | 86 (20%) |  | 57 (32%) | 29 (12%) |  |  |
|  |  |  |  |  |  |  |  |  |  |
| Symptomatic on later test | Yes | 5 (38%) | 136 (45%) | 155 (36%) | 0.20 | 79 (45%) | 76 (30%) | 0.01 |  |
|  | No | 4 (31%) | 87 (29%) | 145 (34%) |  | 47 (27%) | 98 (39%) |  |  |
|  | Missing symptom status | 4 (31%) | 77 (26%) | 127 (30%) |  | 51 (29%) | 76 (30%) |  |  |
|  |  |  |  |  |  |  |  |  |  |
|  |  |  |  |  |  |  |  |  |  |
|  |  |  |  |  |  |  |  |  |  |
| P values calculated by ANOVA/t-test or chi squared test  CO – community onset, HA – healthcare associated. | | | | | | | | | |

| **Supplemental Table 4.** Analysis of positive tests for respiratory viruses during a period of universal admission testing that could have met surveillance criteria for HARVI absent such admission testing. | | | | | |
| --- | --- | --- | --- | --- | --- |
| **Virus** | **No. HARVIs** | **No. collected in definite-HA window (first positive or repeat positive)** | **No. collected in definite-HA window (repeat positive)** | **No. from prior column for which, on admission screen, patient was asymptomatic or had missing symptoms status** | **No. from prior column that were symptomatic on their repeat positive test** |
| ADV | 12 | 19 | 7 | 1 | 0 |
| ccCOV | 7 | 19 | 8 | 2 | 1 |
| HMPV | 4 | 10 | 5 | 1 | 1 |
| FLU | 0 | 6 | 4 | 2 | 0 |
| PIV | 8 | 28 | 18 | 3 | 0 |
| RSV | 3 | 19 | 15 | 1 | 1 |
| REV | 91 | 264 | 150 | 57 | 8 |
| SARS-CoV-2 | 10 | 80 | 56 | 17 | 2 |
| Total | 125 | 427 | 250 | 79 | 12 |
| HARVI – healthcare-associated respiratory viral infection, HA – healthcare-associated, ADV – adenovirus, ccCOV – common cold coronaviruses (HKU1, NL63, 229E, OC43), HMPV – human metapneumovirus, FLU – influenza A/B, PIV – parainfluenza (type 1, 2, 3, or 4), RSV – respiratory syncytial virus, REV – rhinovirus/enterovirus, SARS-CoV-2 – severe acute respiratory syndrome coronavirus 2. Totals do not add up from the individual viruses due to coinfections. | | | | | |

**Supplemental Figure 1**: Diagram demonstrating an example of the onset categories (This example is for respiratory syncytial virus, each virus had unique cut-offs based on minimum and maximum incubation period). Black represents time periods that were excluded from this analysis. Blue represents “present on admission” category, yellow represents “Possible healthcare-associated window”, green represents “definite healthcare-associated window”, and red represents “repeat positive”. During the time this study was conducted patients were required to have an admission screen for respiratory viruses.
